# Influence of the Gut Microflora and of Biliary Constituents on Morphological Changes in the Small Intestine in Obstructive Jaundice

**DOI:** 10.1155/1996/43159

**Published:** 1996

**Authors:** M. Saeed Quraishy, Dawn Chescoe, Jenny Mullervy, Marie Coates, Richard H. Hinton, Michael E. Bailey

**Affiliations:** 1 Microstructural Studies Unit University of Surrey Guildford Surrey GU2 5XH UK; 2 School of Biological Science University of Surrey Guildford Surrey GU2 5XH UK; 3 Department of Surgery Royal Surrey County Hospital Guildford Surrey GU2 5XX UK

## Abstract

Increased amounts of intestinal endotoxin are absorbed in obstructive jaundice. The precise mechanism is not known but the increased absorption may arise from alterations in the luminal contents, in the intestinal flora, in the gut wall or in interactions between all three. To examine the effects of the intestinal flora we have compared the morphological changes in the small intestine in obstructive jaundice in germ free and conventional rats while the effects of bile constituents have been examined by addition of bile constituents to the diet of bile duct ligated rats. Changes in the intestine were examined, histologically, by enzyme histochemistry, and by transmission and scanning electron microscopy. The results showed no differences in response between germ free and conventional rats. Feeding of diets containing bile salts exacerbated the lesion. Feeding of diets containing cholesterol, however, reduced the degree of intestinal changes produced by cholestasis and completely antagonised the increase in damage caused by feeding of bile salts.


*HPB Surgery*, 1996, Vol.10, pp.11–20
Reprints available directly from the publisher
Photocopying permitted by license only

© 1996 OPA (Overseas Publishers Association)
Amsterdam B.V. Published in The Netherlands
by Harwood Academic Publishers GmbH
Printed in Malaysia

### Influence of the Gut Microflora and of Biliary Constituents on Morphological Changes in the Small Intestine in Obstructive Jaundice

M. SAEED QURAISHY, DAWN CHESCOE+, JENNY MULLERVY+, MARIE COATES++, RICHARD H. HINTON++ and MICHAEL E. BAILEY*

*Department of Surgery, Royal Surrey County Hospital, Guildford, Surrey GU2 5XX
+Microstructural Studies Unit
++School of Biological Science, University of Surrey, Guildford, Surrey GU2 5XH

*(Received 19 August 1993)*

Increased amounts of intestinal endotoxin are absorbed in obstructive jaundice. The precise mechanism is not known but the increased absorption may arise from alterations in the luminal contents, in the intestinal flora, in the gut wall or in interactions between all three. To examine the effects of the intestinal flora we have compared the morphological changes in the small intestine in obstructive jaundice in germ free and conventional rats while the effects of bile constituents have been examined by addition of bile constituents to the diet of bile duct ligated rats. Changes in the intestine were examined, histologically, by enzyme histochemistry, and by transmission and scanning electron microscopy. The results showed no differences in response between germ free and conventional rats. Feeding of diets containing bile salts exacerbated the lesion. Feeding of diets containing cholesterol, however, reduced the degree of intestinal changes produced by cholestasis and completely antagonised the increase in damage caused by feeding of bile salts.

KEY WORDS: Gut microflora endotoxin jaundice diet

### INTRODUCTION

There is a high incidence of complications following diagnostic and therapeutic intervention in patients with obstructive jaundice[1,2]. There is considerable evidence to implicate endotoxaemia in the pathogenesis of the renal[3,4], hepatic[5], immunological[6] and coagulative disorders[7] frequently seen in these patients. Endotoxaemia results from an increased absorption[8,9] of luminal endotoxin into the portal circulation and its decreased clearance by the Kupffer cells[10,11] allowing it to spill over into the systemic circulation. However, the nature of the intestinal changes which allow increased absorption of endotoxin during cholestasis are not fully understood.

In theory an increase in endotoxin absorption could arise from alterations in the luminal contents, in the intestinal flora, in the gut wall or interactions between the three. It has been postulated that the absence of bile increases the absorption of endotoxin[9,12] but clinical studies have yielded conflicting results following administration of bile salts[13–15]. Furthermore in a clinical study no alteration in the small bowel flora was found in jaundiced subjects when compared to controls[13].

Earlier work by our group has provided morphological evidence for changes in the intestine of bile duct ligated rats indicative of increased erosion of the microvilli of the enterocytes[1b]. This would seem paradoxical because bile salts are strong detergents and direct administration is known to damage the wall of the intestine[17] so it would be expected that their removal

*Address for correspondence:* Mr. M.E. Bailey, Department of Surgery, Royal Surrey Hospital, Guildford, Surrey GU2 5XX. U.K.

would, if anything, decrease the absorption of high molecular weight materials such as endotoxins. There is hence a possibility that other factors, for example, an alteration in the bacterial flora in the small intestine might pay a role, although no evidence for such a change has been found in human patients. To examine the relative contribution of these factors, we have studied the effects of removing intestinal organisms and of the addition of bile constituents on the damage to the wall of the small intestine which occurs in cholestasis.

### MATERIALS AND METHODS

### *Animals*

Conventional male Lister Hooded rats weighing approximately 250 g were obtained from the University of Surrey Experimental Biology Unit. Unless otherwise mentioned, the rats were fed standard rat diet (CRM diet, Labsure, Manea, Cambs.) and allowed free access to water. The animal rooms were maintained at 22 ± 2°C with a 12h: 12h light: dark cycle.

Male germ free rats (University of Surrey, Experimental Biology Unit) were housed in a pressurised polyvinyl chloride isolator in cages with mesh wire bottoms. Incoming air was filtered through glass wool and bacterial filters. The animals were kept on irradiated rat diet (Labsure, Manea, Cambs) until 24 hours before experiments. As standard rat diets are not endotoxin-free, the rats, from then on, were fed sterile liquid diet (Fortison with Fibre, Cow and Gate, UK) and mixed grains which were washed and baked in oil at 180°C. They were allowed free access to autoclaved water which had been supplemented with vitamins. Sterility was checked every three days by faecal culture and, at termination, by culturing the caecal contents and faeces of all the rats.

### *Surgical Procedures*

This study consists of two separate experiments. The first experiment was designed to compare the response of germ free rats and conventional rats. Each group consisted of 12 animals, 6 of which were subjected to bile duct ligation and 6 to sham operation. The second study investigated the role of bile salts and cholesterol in modulating intestinal damage. The study involved 5 experimental groups each of three animals treated according to the following protocol:-

*Group 1* Sham operation followed by administration of control diet.

*Group 2* Bile duct ligation followed by administration of control diet.

*Group 3* Bile duct ligation followed by administration of a control diet supplemented with 1% bile salts.

*Group 4* Bile duct ligation followed by administration of a control diet supplemented with 1% cholesterol.

*Group 5* Bile duct ligation followed by administration of a control diet supplemented with 1% bile salts and 1% cholestrol.

The bile salt mixture contained cholic acid, deoxycholic acid, chenodeoxycholic acid and lithocholic acid in the same proportions as found in normal rat bile[18]. All procedures were carried out under pentobarbital anaesthesia (Sagatal, May and Baker, Dagenham, UK), a dose of about 6 mg/100g body weight normally being appropriate. Germ free rats were operated on under strict sterile conditions inside the isolators. A 2 cm long upper mid line incision was made and the common bile duct was identified, ligated at two points and divided[19]. In animals undergoing sham ligation, the common bile duct was mobilised and a suture passed under it but not tied, and then removed.

The animals were sacrificed by anaesthetic overdose three weeks after operation. Blood was collected by cardiac puncture. Where appropriate 1ml was placed in endotoxin-free tubes containing 30U of sodium heparin, the remaining blood was aliquoted into lithium-heparin tubes and used for biochemical assays. In experiment I, 1 ml of blood was drawn from the portal vein into endotoxin-free tubes containing 30U sodium heparin. Segments of small intestine were taken from the duodenum (immediately distal to the point of entry of the bile duct), jejunum (measured as 28 cm from the entry of the bile duct) and from distal ileum (5 cm from ileo-caecal junction) and prepared for light and electron microscopy. The caecum was removed for estimation of endotoxin content and, in the case of germ free animals for culture to confirm their microbiological status.

### *Blood Analysis*

Blood collected on the day of the autopsies was analysed by standard techniques for total bilirubin, alanine aminotransferase and alkaline phosphatase on a Falcon autoanalyser (Instrumentation Laboratories, Spokane, WA).

### *Endotoxin Assay*

Endotoxins in portal and systemic blood were measured using a chromogenic limulus amoebycyte lysate test [Coatest Endotoxin, Kabivitrum, Uxbridge, UK] using microtitre plates. Endotoxin levels in faeces were measured by making a suspension of caecal contents in endotoxin free water (Sigma, Poole, Dorset) in a concentration of 10 mg/ml. This was then centrifuged and supernatants collected. Serial dilutions were made to bring the endotoxin levels to within the measurable range of the assay.

### *Microscopy*

Specimens for light microscopy were immediately fixed in neutral buffered 10% formalin. They were then processed, embedded in paraffin and sectioned at 5 μm. Sections were stained routinely with haematoxylin and eosin, by the periodic acid-Schiffs technique (PAS) and with 3% alcian blue stains[20].

Sections for electron microscopy were prepared by an adaptation of the method of Mann *et al*[21]. Small segments of intestine, approximately 1.5 cm long were immediately perfused with a solution of 4% glutaraldehyde buffered with sodium cacodylate (pH 7.4) at room temperature and left overnight in the fixative. They were then washed in 0.1M cacodylate buffer for 24 hours. Small rings, approximately 3 mm long, were cut from the intestine and counterfixed in 2% buffered osmium tetroxide for 2h. They were then dehydrated in a graded series of ethanol and propylene oxide and embedded in Epon 812. Sections were cut, collected on copper grids, counter stained with uranyl acetate[22] and lead citrate[23] and examined.

Specimens for scanning electron microscopy were prepared in the same way up to the completion of dehydration with ethanol. The segments were then opened and immersed in acetone, critically point dried, mounted on aluminium stubs, sputter coated with gold and examined in a Cambridge Instruments scanning electron microscope.

Specimens for enzyme histochemistry were frozen in hexane at −80°C and then stored at −80°C until stained[24]. Staining was carried out within 28 days of autopsies. 10 μm sections were cut in a cryostat. In most cases the sections were stained according to the protocols given by Chayen and Bitensky[24] except that when staining for alkaline phosphatase the sections were incubated in the medium for 3 minutes, and when staining for 5'-Nucleotidase, bromotetramisole was omitted from the incubation medium and replaced by 100 mM L-phenylalanine and staining was done for 5 minutes.

### RESULTS

### *1) Comparison of the Intestinal Effects of Cholestasis in Germ Free and Conventional Rats*

Following bile duct ligation there was a fall in body weight in both germ free and conventional rats. Routine histological examination revealed no alterations in the intestines following ligation of the common bile duct. Examination following staining by the periodic acid-Schiffs technique or with alcian blue showed an increase in size of the goblet cells in the bile duct ligated animals. Consistent with these results, scanning electron microscopy showed an increase in the mucus adherent to the surface of the cells and also showed an increase in desquamating cells and a shortening and irregularity in the microvilli of bile duct ligated animals (Figures 1a, 2a, b). This was confirmed by transmission electron microscopy (Figures 1b, 2c, 2d) which also showed a thickening of the terminal web and hypertrophy of the golgi apparatus in bile duct ligated animals. In addition there was some increase in the incidence of damaged mitochondria in bile duct ligated animals and, in some cells, the mitochondria were markedly condensed. Histochemical staining showed a patchy loss of the brush border enzymes alkaline phosphatase and 5'- nucleotidase consistent with the results of electron microscopy. No difference was observed between germ-free and conventional animals in any of these studies.

### *2) Endotoxin Levels in Germ Free and Conventional Animals*

To confirm the endotoxin poor status of germ free animals we examined the amount of endotoxin in faces and in portal and systemic blood. Very low levels (37.5 ± 2.0 ng/g) of endotoxin was detected in the caecal contents of germ free rats but levels were about 0.05% of the levels (77.7 ± 9.4 μg/g) found in coventional animals. Endotoxin levels in both portal and systemic circulation were below the limits of detection in both SHAM and BDL germ free animals. As microbiological tests confirmed the germ free status of the animals it was assumed that the caecal endotoxin was derived from the diet on which the animals were reared. Conventional animals showed the expected increase in circulating endotoxin following bile duct ligation, changes being greater in the portal vein than in the systemic circulation (Figure 3).

a)

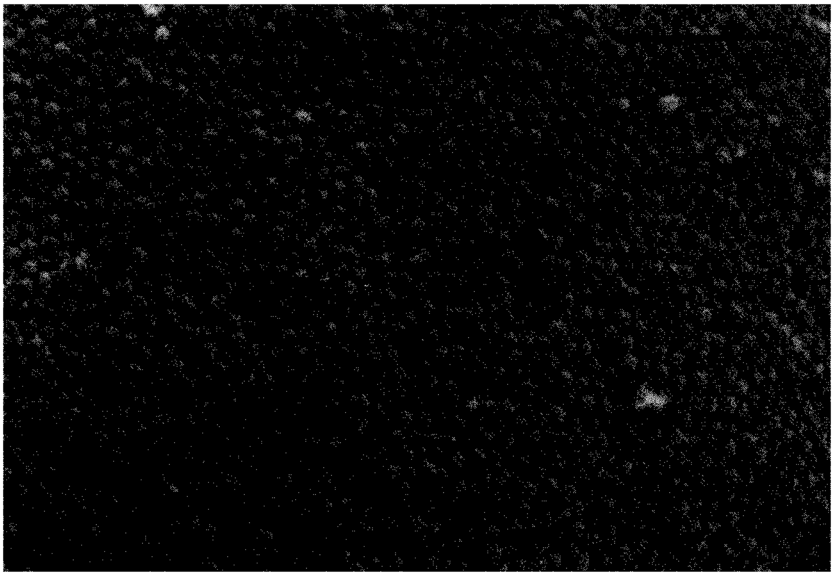

b)

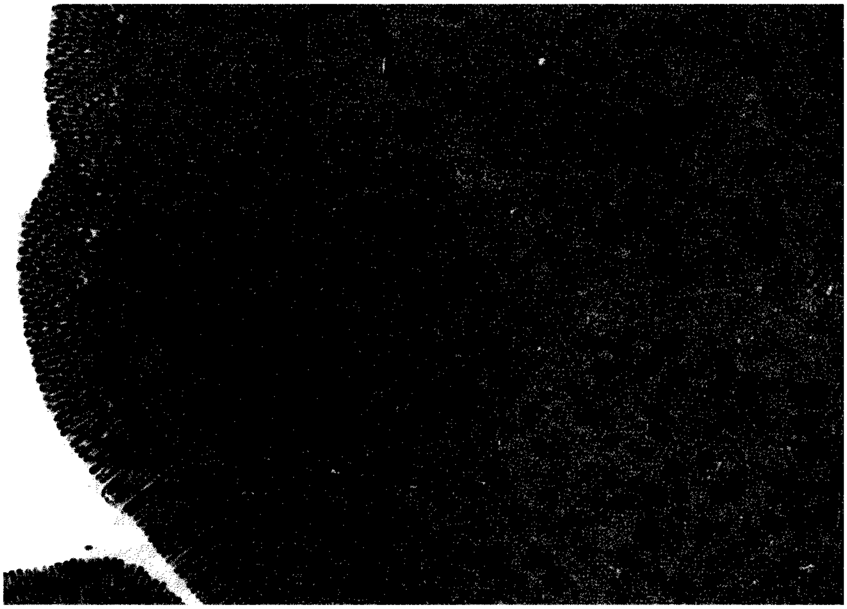

**Figure 1** a) Scanning electron micrograph showing regular arrangement of microvilli in the jejunum of a sham-operated animal, b) Transmission electron micrograph showing normal cell architecture in a sham-operated animal.

### *3) Effects of Bile Salts and Cholesterol on Changes in the Intestinal Morphology Following Bile Duct Ligation*

Administration of bile salts resulted in a marked exacerbation of damage to the enterocytes as assessed both by scanning (Figure 4a) and transmission (Figure 4b) electron microscopy and by histochemical staining. Both damage to the brush border and the golgi hypertrophy were increased. Administration of cholesterol, on the other hand appeared to ameliorate the damage. In bile duct ligated animals fed cholesterol-enriched diets the changes were less marked than in animals not receiving the supplement (Figure 5) although there remained significant difference from control animals. Co-administration of cholesterol prevented the increase in intestinal damage caused by bile salts, the architecture being indistinguishable from that of bile-duct ligated animals receiving no dietary supplements.

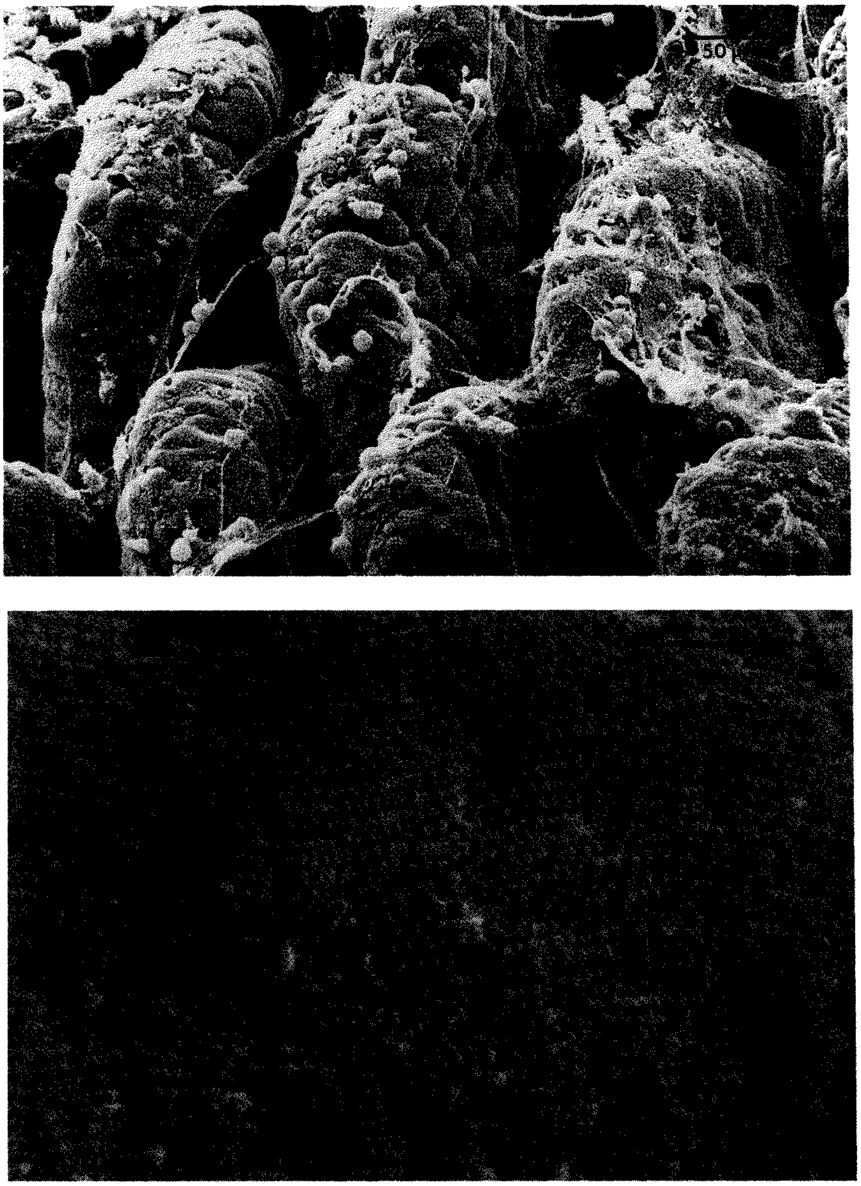

a)

b)

**Figure 2** Electron micrographs of the jejunum in bile duct ligated rats. a) Low magnification scanning electron micrograph showing increased mucus and debris, b) Scanning electron micrograph showing disorganisation of microvilli d) High magnification transmission electron micrograph demonstrating marked Golgi hypertrophy. d) High magnification transmission electron micrograph demonstrating marked Golgi hypertrophy.

### DISCUSSION

Absorption of endotoxin increases in obstructive jaundice[8,9,12] and endotoxaemia has been implicated in the pathogenesis of a number of end organ effects in the presence of cholestasis[3–7]. Endotoxins are also known to potentiate the effects of ischaemic injury[25]. In earlier studies we have reported that the increase in endotoxin absorption is accompanied by morphological changes in the intestine[16] and now present the results of studies designed to determine the pathogenesis of these changes.

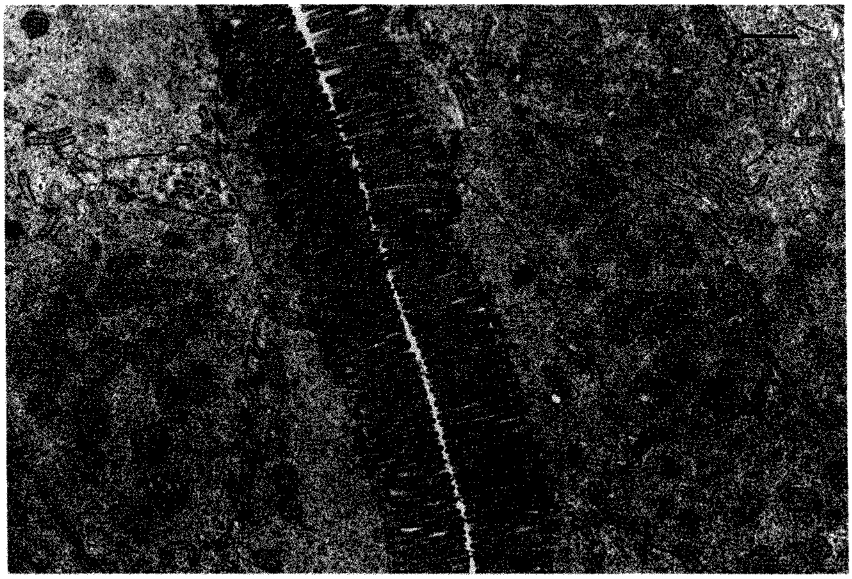

Our results showed that morphological changes in the small intestine during cholestasis were identical in the endotoxin-poor germ free rats and conventional animals falsifying the hypothesis that a significant change in the intestinal flora could explain the alterations to intestinal morphology. This is consistent with the findings of a clinical study where no significant c)

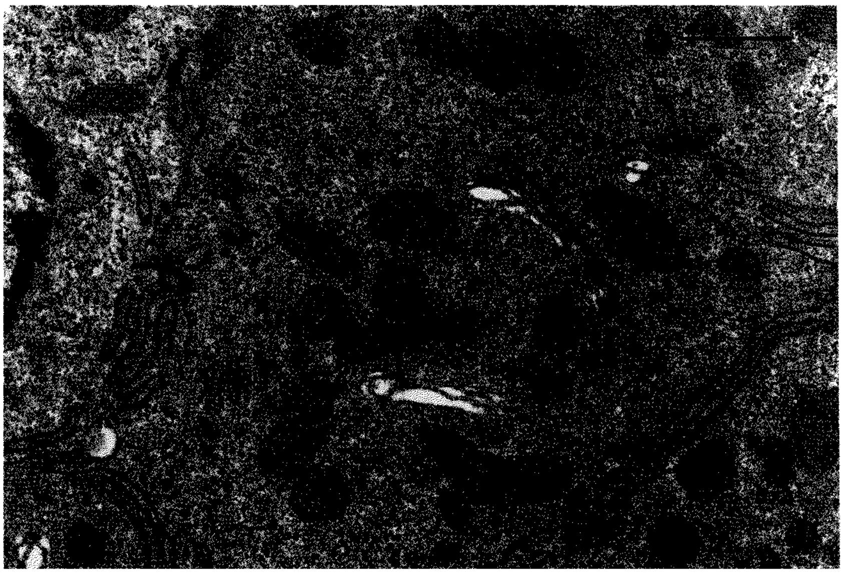

d)

**Figure 2** (*Continued*)

difference of the gut flora was found between controls and patients with obstructive jaundice [13].

As the intestinal changes in cholestasis do not appear to be associated with changes in the bacterial flora it seemed possible that the structural integrity of the small intestine depends, in some way, on factors present in bile Earlier we suggested that the endotoxaemia of cholestasis might be due to increased absorption secondary to changes in the gut wall. Our results strengthen this hypothesis. The high turnover of intestinal epithelial cells results in many transient breaks in the lining through which high molecular materials may pass. The increase in desquamating cells found in bile-duct ligated animals shows that cell turnover has, indeed, increased, creating gaps through which endotoxin and other materials may pass to the subepithelium. It is therefore necessary to determine the pathogenesis of this damage.

Animal experiments have demonstrated that the flow of bile into the intestine is important in controlling endotoxaemia[26] and in decreasing the mortality from infection[27]. Both clinical and experimental studies have

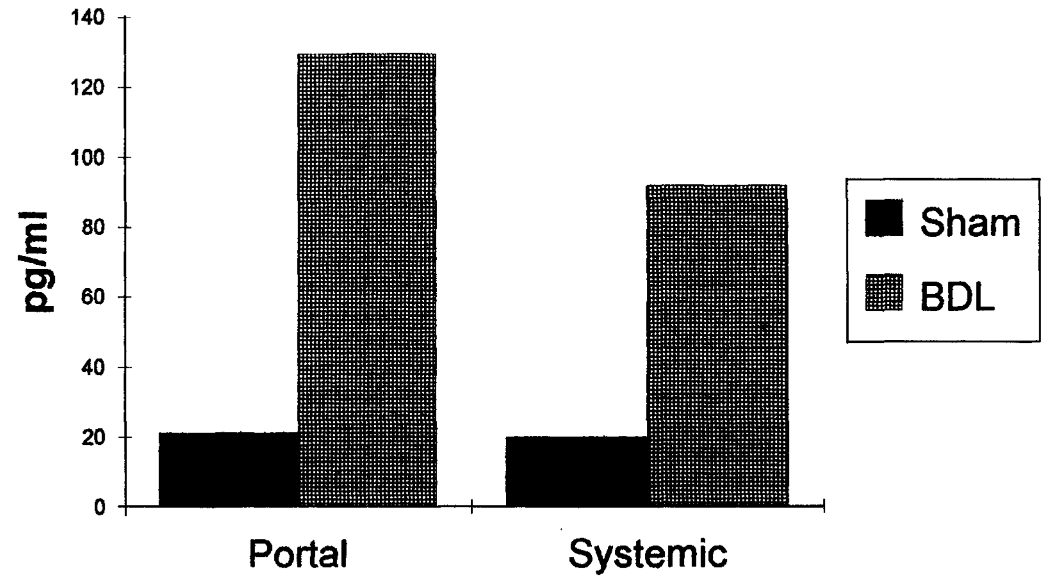

**Figure 3** Figure showing the endotoxon levels in portal and systemic blood from sham-operated and bile duct ligated rats. Each block represents the mean of determinations from 6 animals.

shown that bile salt administration protects against the systemic effects of cholestasis[9,13–14]. Our results, however, show clearly that, as expected from studies on non-jaundiced animals[17,28], bile salts exacerbate the intestinal damage resulting from cholestasis. This drew attention to other bile components. Most of the cholesterol required by the body either derives from the diet or is synthesised in the liver. It therefore appeared possible that biliary cholesterol is required for the maintainance of the enterocytes. This hypothesis was examined and it was found that cholestrol supplementation of diet did indeed ameliorate the damage.

The environment in which intestinal enterocytes exist is, inevitably, extremely hostile. Two factors assist in maintaining the epithelial barrier. Firstly there is rapid turnover of the enterocytes and secondly a)

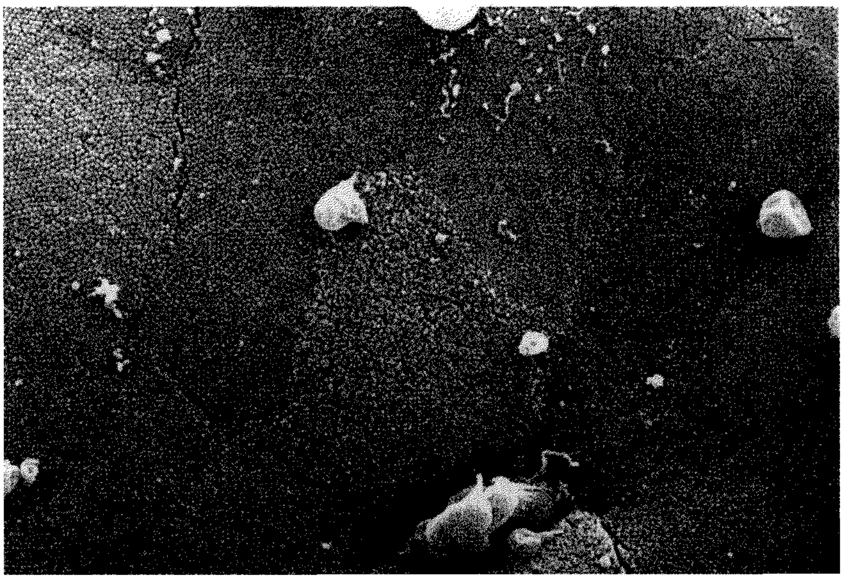

**Figure 4** a) Low magnification scanning electron micrograph showing increased surface damage in the jejunum of a bile duct-ligated rat fed bile salts b) Transmission electron micrograph showing marked erosion of the microvilli and disruption of the intracellular organelles in a bile duct-ligated rat fed bile salts.

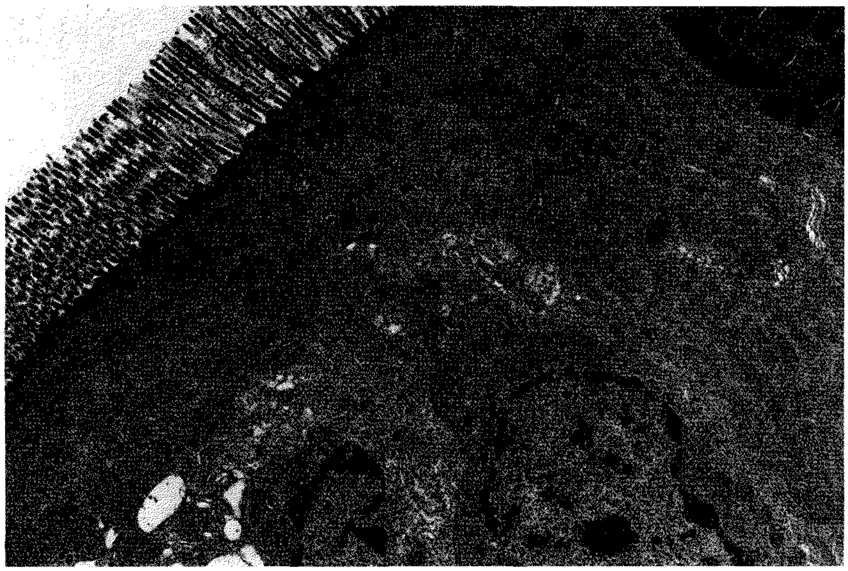

b)

**Figure 4** (*Continued*)

enterocytes also have considerable capacity for self-repair as evidenced by the well-developed rough endoplasmic reticulum and golgi apparatus which supply proteins to replace those eroded from the brush border[29]. Both formation of new cells and repair to the brush border will require continuous supplies of cholesterol. The changes observed in our experiments indicate increased erosion. This is shown directly by the irregularities in the brush border and indirectly by the compensatory hypertrophy of the golgi apparatus. The close connection between the two phenomena is emphasised by the effects of the administration of bile salts. These, as would be expected from their detergent properties, exacerbated damage to the brush border and not only increased hypertrophy of the golgi apparatus but also caused some increase in the rough endoplasmic reticulum.

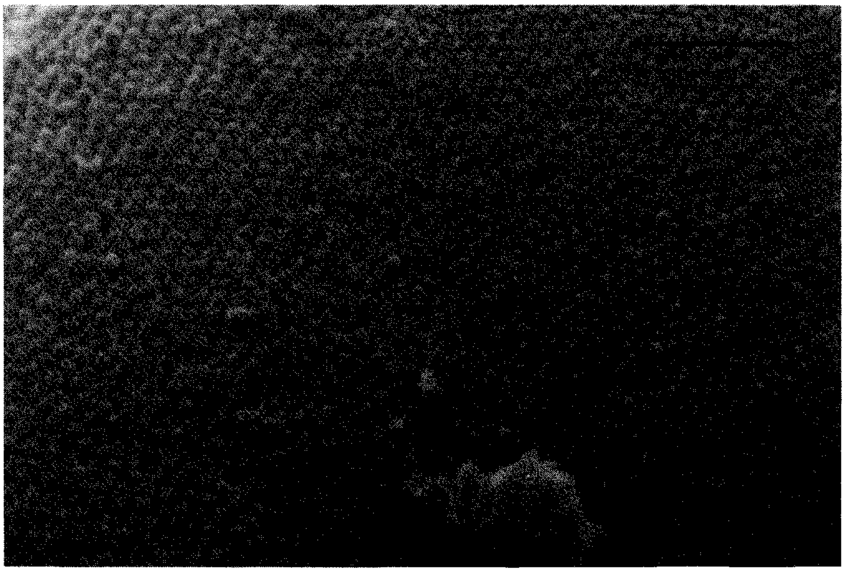

It would thus appear that, in the absence of bile, a)

**Figure 5** Electron micrographs showing that damage to the microvilli in bile duct-ligated is ameliorated following administration of cholesterol. a) Scanning electron micrograph b) Transmission electron micrograph b) 

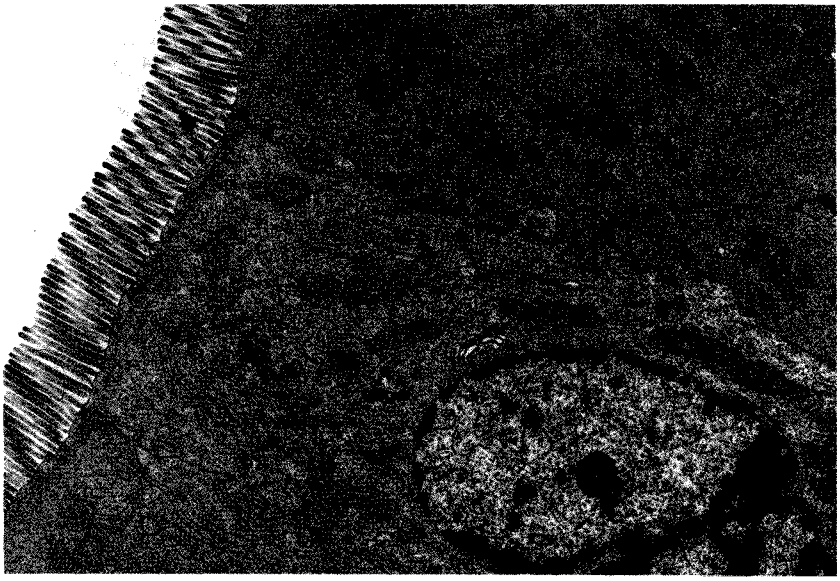

**Figure 5** (*Continued*)

**Table 1** a) Liver function tests in conventional and germ free animals following either sham operation or bile duct ligation. Results are presented as mean ± SEM for groups of 6 animals.

| | | Bilirubin µmol/L | Alkalime phosphatase | Alanine amino-transferase |
|---|---|---|---|---|
| | | | IU/l | IU/l |
| Conventional | Sham | 3.7 ± 0.2 | 143 ± 11 | 105 ± 9 |
| | BDL | 234 ± 12 | 908 ± 71 | 247 ± 19 |
| Germ Free | Sham | 2.0 ± 0.5 | 104 ± 13 | 93 ± 7 |
| | BDL | 138 ± 14 | 1178 ± 168 | 256 ± 11 |

b) Liver function tests on conventional rats following either sham operation, bile duct ligation (BDL) or bile duct ligation followed by feeding of diets supplemented with cholesterol, bile salts or both. Results are presented as mean ± SEM for groups of 3 animal.

| | Bilirubin µmol/L | Alkalinie phosphatase | Alanine amino-transferase |
|---|---|---|---|
| | | IU/l | IU/l |
| Sham operated | 2 ± 0 | 137 ± 13 | 106 ± 7 |
| BDL | 122 ± 7 | 900 ± 57 | 308 ± 58 |
| BDL+Cholesterol | 167± 14 | 1235± 168 | 453± 73 |
| BDL+Bile Salts | 238 ± 73 | 1368 ± 254 | 413 ± 21 |
| BLD+Bile Salts+Cholesterol | 164 ± 12 | 1303 ± 282 | 291 ± 57 |

there is increased erosion of the brush border of enterocytes and a resultant increase in cell turnover. Our results suggest that cholesterol may contribute to the protective action of bile. Cholesterol is essential to the stability of the plasma membrane. The high rate of turnover of enterocytes and their extremely convoluted plasma membrane will result in a considerable demand for cholesterol and it would appear likely that this is satisfied, at least in part, by direct utilisation of dietary cholesterol and cholesterol delivered to the intestinal lumen via the bile duct. Standard rat laboratory diets are low in cholesterol so it is possible that the absence of biliary cholesterol may affect the assembly of the brush border or limit its ability for repair. This hypothesis offers a possible explanation of the differences in damage along the length of the small intestine, because the duodenum and proximal part of the jejunum will be protected by cholesterol in the diet while the cholesterol content of chyle in the ileum will be boosted by cells eroded higher up the intestine.

### ACKNOWLEDGEMENTS

We acknowledge with gratitude the facilities and advice extended to us by Dr. S.C. Price in the Robens Institute of Health and Safety, the invaluable help and advice on histochemical techniques provided by Dr. J. Chayen and Dr. L. Bitensky of the Cellular Pharmacology Unit of the Robens Institute and the skill of Ms V. Ronaasen in the breeding, maintainance and handling of the germ free rats. Preliminary reports of a part of these results has been presented at the 4th World Hepatopancreatobiliary Congress in Hong Kong.

### REFERENCES

1. Pain, J.A., Cahill, C.J., Bailey, M.E. (1985) Perioperative complications in obstructive jaundice: therapeutic considerations. *Br. J. Surg.*, **72**, 942–945.
2. Gillen, P. (1986) Pell ALG Failure to improve survival by improved diagnostic techniques in patients with malignant jaundice. *Br. J. Surg.*, **73**, 631–633.
3. Wardle, E. N. (1970) Wright NA Endotoxin and acute renal failure associated with obstructive jaundice, *Br. Med., J.* **4**, 472–474.

4. Wilkinson, S.P., Moody, H., Stamatakis, J.D., Kakkar, V.V., Willams, R. (1976) Endotoxaemia and renal failure in cirrhosis and obstructive jaundice, *Br. Med. J.* **2**, 1415–1418.
5. Utili, R., Abernathy, C.O., Zimmerman, H.J. (1976) Cholestatic effects of Escherichia coli endotoxin on the isolated perfused rat liver. *Gastroenterology.* **70**, 248–253.
6. Greve, J.W., Gouma, D.J., Soeters, P.B., Buurman, W.A. Supperession of cellular immunity in obstructive jaundice is caused by endotoxins: A study with germ free rats. *Gastroenterology* (1990) **98**, 478–485.
7. Hunt. D.R., Allison, M.E.M., Prentice, C.R.M., Blumgart L.H. (1982) Endotoxaemia disturbance of coagulation and obstructive jaundice. *Am. J. Surg.*, **144**, 325–329.
8. van Deventer, S.J.H., ten Cate. J.W., Tytgat, G.N.J. (1988) Intestinal endotoxaemia. *Gastroenterology*, **94**, 825–831.
9. Bailey, M.E. (1976) Endotoxin, bile salts and renal function in obstructive jaundice. *Br. J. Surg.*, **63**, 774–778.
10. Drivas, G., James, O., Wardle, N. (1976) Study of reticuloendothelial phagocytic capacity in patients with cholestasis. *Br. Med. J.* **1**, 1568–1569.
11. Holman, J.M., Rikkers, L.F. (1982) Biliary obstruction and host defense failure *J. Surg., Res.*, **32**: 208–213.
12. Koscar, L.T., Bertok, L., Varteresz, V. (1969) Effect of bile acids on the intestinal absorption of endotoxin in rats. *J. Bacteriol.*, **100**, 220–223.
13. Cahill, C.J. (1983) Prevention of postoperative renal failure in patients with obstructive jundice-the role of bile salts. *Br. J. Surg.*, **70**, 590–595.
14. Evans, H.J.R., Torrealba, V., Hudd, C., Knight, M. (1982) The effect of preoperative bile salt administration of post operative renal function in patients with obstructive jaundice. *Br. J. Surg.*, **69**, 706–708.
15. Thompson, J., Cohen, J., Blenkharn., J., Blumgart, L.H. (1986) Randomised clnical trial of ursodeoxycholic acid in obstructive jaundice. *Br. J. Surg.*, **73**, 634–636.
16. Singh, S., Bailey, M.E. (1990) A scanning and transmission electron microscopic study of small bowel in experimental cholestasis in the rat. *HPB Surg.*, **2** (Suppl) 479.
17. Gracey, M., Papadimitrou, J., Burke, V. (1973) Effects on small intestinal function and structure induced by feeding a denconjugated bile salt. *Gut.*, **14**, 519–528.
18. Rahman, K., Billington, D. (1984) Effect of chenode-oxycholate feeding upon the biliary output of plasma membrane enzymes in the rat. *Biochem. Pharmacol.* **33**, 2231–2238.
19. Lee, E. (1972) The effect of obstructive jaundice on the migration of reticulo-endothelial cells and fibroblasts into early experimental granulomata. *Br. J. Surg.*, **59**, 875–877.
20. Bancroft, J.D., Stevens, A. (1982) Theory and Practice of Histological Techniques ed 2 Churchill-Livingstone, London .
21. Mann, A.H., Price, S.C., Mitchell, F.E., Grasso, P., Hinton, R.H., Bridges, J.W. (1985) Comparison of the short term effects of di(2-ethylhexyl)phthalate, di(n-hexyl)phthalate and di(noctyl) phthlate in rats. *Toxicol. Appl. Pharmacol.*, **77**, 116–132.
22. Stempak, J.G., Ward, R.T. (1964) An improved staining method for electron microscopy. *J. Cell. Biol.* **22**:697–701.
23. Reynolds, E.S. (1964) The use of lead citrate at high pH as an electron opaque strain in electrom microscopy. *J. Cell. Biol.*, **17**, 208–212.
24. Chayen, J., Bitensky, L. (1992) Practical Histochemistry 2nd Edition. *J. Wiley Chichester.*
25. Zagar, R.A. (1986) Escherichia coli endotoxin injections potentiate experimental ischaemic renal injury. *Am. J. Physiol.*, **251**. F998–994.
26. Gouma, D.J., Coelho, J.C.U., Fisher, J.D., Schlegel, J.F., Li, Y.F., Moody, F.G. (1986) Endotoxaemia after relief of biliary obstruction by internal and external drainage in rats. *Am. J. Surg.*, **151**, 476–479.
27. Gouma, D.J., Coelho, J.C.U., Schlegel, J.F., Li, Y.F., Moody, F.G. (1987) The effect of preoperative internal and external biliary drainage on mortality of jaundiced rats. *Arch. Surg.*, **122**: 731–734.
28. Beeken, W.L., Roessner, K.D., Krawitt, E.L. (1974) Effect of deoxycholate, indole, and endotoxin on organ cultures of rabbit jejunum. *Gastroenterology.*, **66**, 998–1004.
29. Weiss, L. (ed) (1988) Cell and tissue biology: A textbook of histology. Urban and Schwarzenberg, Baltimore.

